# Study of Tumor-Infiltrating Lymphocytes in Breast Carcinoma and Their Association With Pathological and Prognostic Factors and Pathological Tumor-Node-Metastasis (pTNM) Staging

**DOI:** 10.7759/cureus.66657

**Published:** 2024-08-11

**Authors:** Abhishek Gupta, Smita Chandra, Kanika Munish

**Affiliations:** 1 Pathology, Gautam Buddha Chikitsa Mahavidyalaya, Dehradun, IND; 2 Pathology, Himalayan Institute of Medical Sciences, Dehradun, IND

**Keywords:** tumor, til (tumor infiltrating lymphocytes), stromal til, prognosis, breast carcinoma

## Abstract

Objectives: Breast carcinoma is the second most frequent type of cancer globally, with an estimated 2.08 million new carcinoma cases identified in 2018. Breast cancer prognosis is influenced by a number of variables, including the patient’s age, morphological variant, stromal inflammatory reaction, elastotic, fibrotic focus, lymphovascular emboli, recurrence of tumor, etc. Recently, the morphological evaluation and extent of tumor-infiltrating lymphocytes (TIL) have also been studied in breast cancer. An attempt is being made to understand the role of TIL in determining the prognostication of carcinoma breast. Thus, the goal of the current academic study is to assess TIL in breast carcinoma.

Materials and method: The study was performed at a medical institution’s pathology department, which covered newly diagnosed cases of infiltrating ductal carcinoma of the breast on histopathology during the January to December 2019 time frame. The gross and hematoxyline-eosin-stained paraffin sections were studied for histopathological examination.

Results: The study included 50 cases of infiltrating ductal carcinoma of the breast with a female-to-male ratio of 24:1. Stromal TIL was negative (0-10%) in 12 cases, while was positive (11-100 %) in 38 cases. The results of the receiver operating characteristic (ROC) curve study indicated that the specificity was 70.7% and the sensitivity was 85.3% when the cutoff of stromal TIL <11% was used to predict the live status of patients.

Conclusion: Stromal TIL is an important parameter that must be reported in breast carcinoma cases. Positive stromal TIL shows a statistically significant difference with pathological tumor-node-metastasis (pTNM) staging, tumor laterality, size of the tumor, and involvement of nipple and areola.

## Introduction

Breast carcinoma is the second most frequent type of cancer globally, with an estimated 2.08 million new carcinoma cases identified in 2018. Worldwide the incidence of breast carcinoma is 25.1%, and the age age-standardized rate is 46.3 per 100,000 women. The incidence is even higher in countries such as India, with 27.7% incidence in females, and the age-standardized rate of 24.7 per 100,000. The estimated mortality rate due to carcinoma breast is recorded at 23.5% [1]. Breast cancer prognosis is influenced by a number of variables, including patient’s age, pregnancy, use of oral contraceptives, time of diagnosis of carcinoma, gross size, site of tumor, morphological variant, grade of tumor, tumor margin, tumor necrosis, stromal inflammatory reaction, elastotic, fibrotic focus, lymphovascular emboli, metastasis to axillary lymph nodes, TNM (tumor/node/metastasis) staging, surgical margins, and recurrence of tumor [2]. Recently, it has been observed that there is a closed interaction between the epithelial carcinoma cells and the stromal cells, which play a vital role in the growth of the various carcinomas. Immune cell infiltration, particularly anti-tumor type 1 lymphocyte infiltration, has been associated with improved outcomes in colon, ovarian, and lung carcinomas [3-5]. Through the release of cytokines and the activation of "antigen-presenting cells" (APCs), CD4+ T-helper 1 (Th1) cells promote antigen presentation, which may be associated with favorable patient survival [6]. Conversely, type 2 CD4+ T-helper cells (Th2), such as Forkhead box P3 (FOXP3) CD4+ regulatory T-cells, help promote the formation of B lymphocytes and block the function of cytotoxic T lymphocytes, which in turn triggers an anti-inflammatory immune response that may accelerate the growth of tumors [7]. Recently, the morphological evaluation and extent of tumor-infiltrating lymphocytes (TIL) have also been studied in carcinoma breast. An attempt is being made to understand the role of TIL in determining the prognosis of breast cancer [8]. In addition to prognosis, the TIL may be associated with the treatment response of breast carcinoma [9]. Thus, the goal of the current study was to assess T.I.L. in breast cancer. It was also intended to analyze T.I.L. with prognostic factors in this carcinoma.

## Materials and methods

This is a prospective observational study. The study was performed at the Department of Pathology at the Himalayan Institute of Medical Sciences, Dehradun, Uttarakhand, for the duration of 12 months. Fifty newly diagnosed cases, with infiltrating ductal carcinoma of the breast by histopathology from January 2019 to December 2019, were included in the study. The cases age ranged from 27 to 81 years old. An informed consent was taken by all the study participants. The study was approved by the institutional review board (SRHU/HIMS/ETHICS/2020/136). The patients diagnosed with core and trucut needle biopsies for infiltrating ductal carcinoma breast are included, and all diagnosed cases who received treatment including surgery, chemotherapy, or radiotherapy were excluded from the study.

Once the patient was diagnosed with carcinoma breast after the relevant history, examination, and pertinent radiological, laboratory investigations by the Department of Surgery, Himalayan Institute of Medical Sciences, Dehradun, Uttarakhand, after the planned surgery, the mastectomy sample was sent to the Department of Pathology, Himalayan Institute of Medical Sciences, Dehradun. The sample was sent in a container containing 10% formalin, completely submerged. After the sample is received grossing and processing of the sample according to standard operating procedure was done. Specifically, 4-5 microns thickness paraffin block sections were made with the help of microtome and stained by hematoxylin and eosin. All stained sections were then studied for breast carcinoma classification, associated histo-morphological features, grading, and TNM staging according to the WHO classification of tumor of the breast [10]. The lymph vascular emboli, necrosis, perineurial invasion, calcification, and lymph node metastasis were also studied. Microscopic evaluation of TIL on hematoxylin and eosin stain sections was done according to the recommendations of the International TIL Working Group 2014 [11]. Each case was followed up for a minimum period of 12 months, and their survival was recorded.

The collected data were prepared into an Excel sheet (Microsoft® Corp., Redmond, WA), and the data were analyzed using the Statistical Product and Service Solutions (SPSS, version 23; IBM SPSS Statistics for Windows, Armonk, NY) software. The relationship between categorical variables was ascertained using the student T-test and Pearson's correlation coefficient. The p value =< 0.05 was considered statistically significant. The receptor operating characteristic (ROC) curve analysis was used to assess how well TIL performs as a diagnostic tool for predicting the case status as alive.

## Results

The study comprised 50 cases of infiltrating ductal carcinoma of the breast with a female-to-male ratio of 24:1. The mean age was found 53.8 and the median age of 56. The highest percentage of cases (26%) were found in the age ranges of 31-40 and 61-70 years old. Of these, only one case had a positive family history of breast cancer.

Table 1 reports the gross and histological findings in patients with breast cancer. Tumor size varies from 2 cm to 5 cm showing the maximum number of cases, 64% (n=32). Lympho-vascular emboli were present in 82% of cases, and lymph node metastasis was seen in 60% of cases. According to pathological TNM staging, stage II cases accounted for the majority of cases (54%).

**Table 1:** Histopathological characteristics and staging of breast carcinoma cases

Pathological characteristics	Number of cases (percentage)
Laterality	
Right breast	29 (58%)
Left breast	20 (40%)
Bilateral	1 (2%)
Tumor Size	
<2 cm	2 (4%)
2-5 cm	32 (64%)
>5 cm	16 (32%)
Modified Richardson-Bloom Score	
3-5 (Grade I)	0
6-7 (Grade II)	37 (74%)
8-9 (Grade III)	13 (26%)
Intratumoral DCIS (>25%)	15 (30%)
Extratumoral DCIS (>10%)	3 (6%)
Calcification	3 (6%)
Lymphovascular emboli	41 (82%)
Perineural invasion	9 (18%)
Lymph node metastasis	30 (60%)
pTNM Stage	
Stage I	1 (2%)
Stage IIA	16 (32%)
Stage IIB	11 (22%)
Stage IIIA	10 (20%)
Stage IIIB	5 (10%)
Stage IIIC	3 (6%)
Stage IV	4 (8%)

Stromal TIL was negative (0-10%) in 12 cases, while positive (11-100 %) in 38 cases. Table 2 shows the correlation between stromal TIL with gross examination, histopathological, and prognostic factors in breast cancer. It also shows that there was a statistically significant difference between stromal TIL and tumor laterality, size, involvement of nipple and areola, and pathological T stage. However, between stromal TIL and pathological TNM staging, modified RB score, and intra- and extra-tumoral ductal carcinoma in situ (DCIS), there was no statistically significant difference.

**Table 2:** Association of stromal TIL with gross examination and histopathological and prognostic factors in breast carcinoma n - Number of cases; TIL - Tumor-infiltrating lymphocytes; RB score - Bloom Richardson score; DICS- Ductal carcinoma in situ

Characteristics	Number of cases (n) showing stromal TIL positivity (11-100%)	Number of cases (n) showing Stromal TIL negativity (0-10%)	p value
Tumor Side			0.038
Right	27	4	
Left	11	8	
Tumor size			0.041
<2 cm	0	2	
2-5 cm	24	8	
>5 cm	14	2	
Nipple and Areola			0.048
Unremarkable	35	8	
Retracted	3	3	
Ulcerated	0	1	
RB Score			0.708
Score 6	18	6	
Score 7	10	3	
Score 8	6	3	
Score 9	4	0	
Intratumoral DCIS			0.304
<25%	25	10	
>25%	13	2	
Extratumoral DCIS	3	0	1
Microcalcification	2	1	1
Lymphovascular emboli	30	11	0.425
Perineural invasion	7	2	1
Lymph node metastasis	6	4	0.225
Pathological T Stage			0.015
1	0	1	
1c	0	1	
2	24	6	
3	12	1	
4a	0	1	
4b	2	2	
Living Status (After 12 Months)			1
Alive	31	10	
Dead	7	2	
Recurrent Status of Tumor (Until 12 Months)			1
Recurrent	6	2	
Not recurrent	2	0	

**Figure 1:**
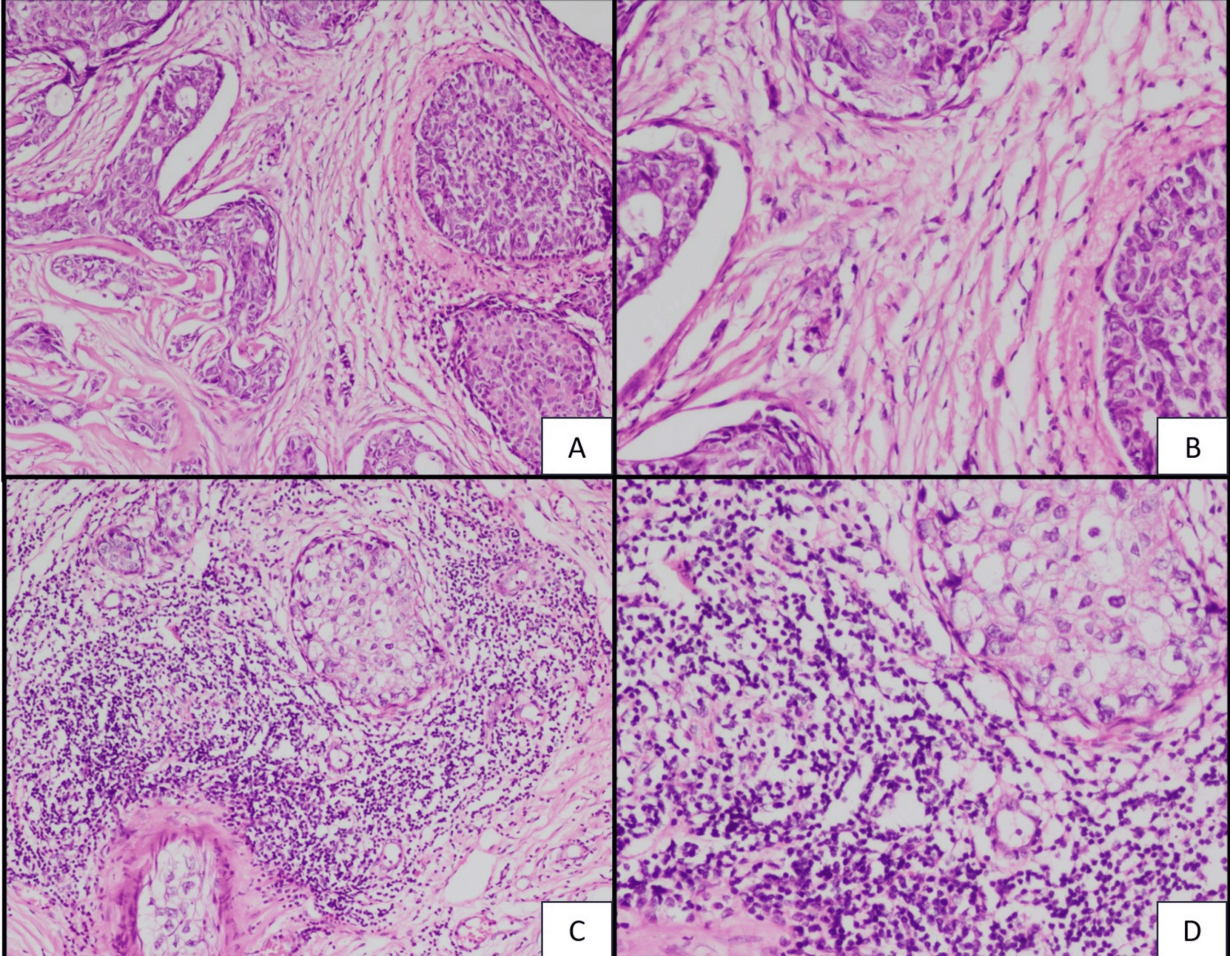
Histopathological section showing stromal TIL expression in breast carcinoma (A, B) (Negative: 0-10%) (H&E, x4 and x40). (C, D) (Positive: 11-100%) (H&E, x4 and x40)

Figure 1 presents the expression of tumor-infiltrating lymphocytes in the stroma of breast carcinoma cases.

**Figure 2:**
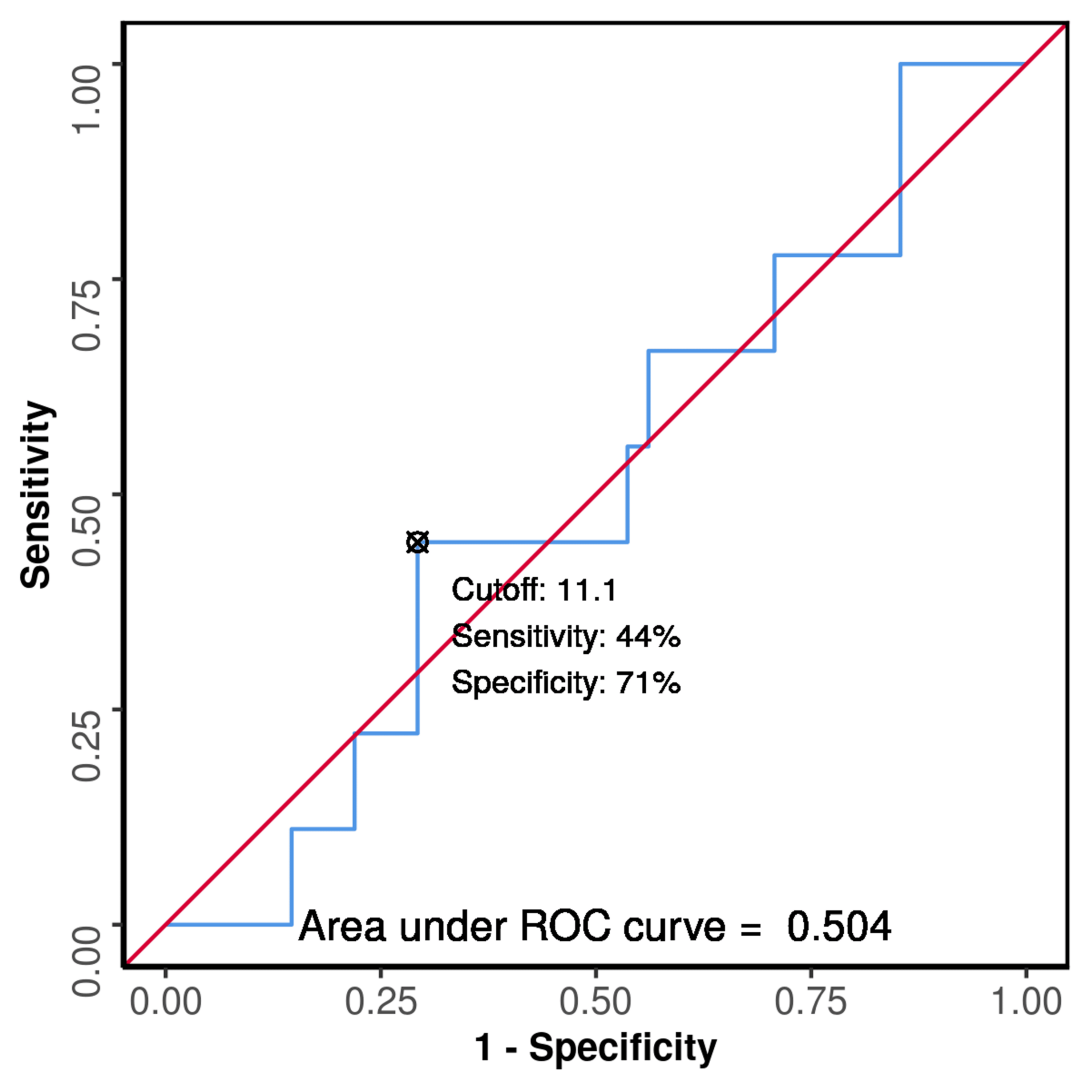
ROC curve analysis showing the diagnostic performance of stromal TIL (%) in predicting the living status of cases (n = 50) ROC - Receiver operating characteristic

Figure 2 and Table 3 show the receiver operating characteristic(ROC) curve analysis indicating that, at the cutoff of stromal TIL <11% for determining the survival status of cases, the specificity was 70.7%, sensitivity was 44.4%, negative predictive value was 85.3%, and positive predictive value was 25% with a diagnostic odds ratio of 1.93.

**Table 3:** ROC curve analysis showing the diagnostic performance of stromal TIL (%) in predicting the living status of cases (n = 50) ROC - Receiver operating characteristic

Parameter	Value (95% CI)
Cutoff (p value)	≤ 11.1 (0.980)
AUROC	0.504 (0.3-0.708)
Sensitivity	44.4% (14-79)
Specificity	70.7% (54-84)
Positive Predictive Value	25.0% (7-52)
Negative Predictive Value	85.3% (69-95)
Diagnostic Accuracy	66.0% (51-79)
Positive Likelihood Ratio	1.52 (0.64-3.63)
Negative Likelihood Ratio	0.79 (0.42-1.46)
Diagnostic Odds Ratio	1.93 (0.44-8.47)

## Discussion

In India, the incidence of breast carcinoma in females is 27.7%, with an age-standardized rate of 24.7 per 100,000 females and a five-year prevalence rate of 32.2%. Breast carcinoma is the most frequent cause of cancer deaths in females of less developed regions, constituting 14.3% of all cancer deaths. Worldwide, the mortality rate of breast cancer in females is 15%, while in India, it is 23.5% [1].

In the present study, 50 cases of breast carcinoma were studied for one year. The present study found that breast carcinoma was more on the right side than the left side, which is contrary to that of Nigam et al. in which the left laterality was found to be more common [12].

In the present study, 64% of cases had tumor size between 2 and 5 cm, and the mean gross tumor size was 4.55±1.76 cm. The Bloom-Richardson system was introduced to grade breast carcinoma cases. Commonly, the Nottingham modification of the Bloom-Richardson system is the most commonly followed system that uses three parameters, namely, tubule formation, nuclear pleomorphism, and mitotic activity [13]. Grading is done exclusively on the invasive component of the tumor [14]. In the present study, 50 cases of breast carcinoma were graded according to the Nottingham modification of the Bloom-Richardson system. Among them, 56% (n=28) cases were of Grade II showing stromal TIL positivity, while 18% (n=9) of Grade II showing stromal TIL negativity.

Lympho-vascular invasion (LVI) is a known marker of a poor prognosis and can predict a worse outcome for patients with invasive breast cancer. LVI may also be used as an indicator of aggressive behavior and the metastatic ability of the primary malignancy [15-17]. Compared to other prognostic factors, perineural invasion has received much less attention in the literature. Some studies have reported perineural invasion in the form of the direct infiltration of nerves [18]. In the present study, LVI was present in 60% of cases showing stromal TIL positivity, while 22% showed negative stromal TIL; the perineural invasion was seen in 14% of cases showing positive stromal TIL, and 4% showed stromal TIL negativity.

In breast carcinoma, T and B lymphocyte-mediated immunity offers the essential framework for potent and long-lasting anti-tumor responses [7]. Tumor infiltration by cytotoxic CD8+ T cells is thought to be closely related to patient survival and treatment response [19]. Stromal TILs are distributed throughout the stroma between the carcinoma cells and do not directly engage with the carcinoma cells; on the other hand, intratumoral TILs are characterized as lymphocytes in tumor nests that have cell-to-cell contact, with no intervening stroma and interact directly with the carcinoma cells [11].

In the present study, stromal TIL was evaluated, and 76% of cases showed a positive TIL percentage. The International TIL Working Group's recommendations regarding the percentage of TILs served as the foundation for the TIL assessment. The working group agreed that scoring TILs as a continuous variable could yield more biologically relevant information since it will enable more precise statistical studies that can then be grouped based on various thresholds [11].

In the present study, it was also found that stromal TIL was statistically significantly positive in patients with right-side breast carcinoma and tumor size of more than 2 cm. The study finding suggests that a positive stromal TIL is linked to larger tumor size in breast cancer since tumor size is considered to be a significant prognostic factor.

The largest percentage of breast carcinoma cases presented were found to be in the pT2N0Mx stage (30%). Large tumor diameter was assessed based on the T stage in most of the studies and was considered to be among the most significant factors influencing patient survival. The larger diameter of the tumor also affects lymph node metastasis [13]. In the present study, the percentage of patients with pathological stages T2, T3, and T4 were 60%, 26%, and 10%, respectively. The pT2 stage showed the highest percentage of tumor metastasis (30%). It was discovered that positive stromal TIL corresponds statistically significantly to the pathological T stage.

The current study had some limitations, which included a small patient group with a shorter follow-up period. This could be the cause of the uneven distribution of patients in certain subgroups, which skewed the statistical analysis. Furthermore, tumor heterogeneity may result in an improper TIL assessment when determining a breast cancer prognosis.

## Conclusions

An essential parameter that needs to be assessed and reported in patients with breast carcinoma is stromal TIL. There is a statistically significant difference between positive stromal TIL and pTNM staging, tumor laterality, size of the tumor, and involvement of nipple and areola. In order to determine the precise function of TIL as a prognostic marker in breast carcinoma, further extensive studies with longer follow-ups are recommended. Thus, it might be crucial in cutting-edge targeted immunotherapy for the treatment of breast carcinoma.
